# Fenestrated endovascular aneurysm repair addressing para-aortic ulcers in the setting of tuberculous aortitis

**DOI:** 10.1016/j.jvscit.2026.102282

**Published:** 2026-04-28

**Authors:** Christopher C. Fawaz, Saman Qadri, Mira T. Tanenbaum, Shawn Sarin, Salim Lala

**Affiliations:** a Department of Surgery, The George Washington University School of Medicine and Health Sciences, Washington, DC; b Division of Interventional Radiology, Department of Radiology, The George Washington University Hospital, Washington, DC; c Division of Vascular Surgery, Department of Surgery, The George Washington University School of Medicine and Health Sciences, Washington, DC

**Keywords:** FEVAR, PAUs, Tuberculosis aortitis, Rifampin-soaked graft

## Abstract

Fenestrated endovascular aneurysm repair (FEVAR) has become a preferred approach for complex aortic pathology involving the visceral vessels, particularly in patients at high risk for open repair. Tuberculosis aortitis is rare, with few documented cases involving the visceral aorta. This case report describes a 68-year-old man with a history of bladder cancer undergoing immunotherapy who developed penetrating aortic ulcers secondary to suspected tuberculosis aortitis and underwent successful laser FEVAR. This case highlights the feasibility and efficacy of laser FEVAR for complex aortic disease such as tuberculosis aortitis and demonstrates its role as a life-saving strategy in high-risk patients.

Paravisceral penetrating aortic ulcers (PAUs) and saccular aneurysms carry a high rupture risk, requiring urgent intervention. In the presence of malignancy, retroperitoneal inflammation, or infection, open surgical repair (OSR) carries prohibitive mortality.^1^, ^2^, ^3^, ^4^, ^5^ Fenestrated endovascular aneurysm repair (FEVAR) offers a minimally invasive alternative that preserves visceral perfusion in high-risk OSR candidates.^6^ Although FEVAR carries a risk of graft infection, prolonged antibiotic therapy has been shown to mitigate this risk. Tuberculosis aortitis is rare, particularly in the visceral aorta; treatments range from OSR to EVAR combined with medical therapy.^7^, ^8^, ^9^

This case report presents a 68-year-old man with bladder cancer undergoing intravesical Bacillus Calmette-Guérin therapy who developed para-aortic lymphadenopathy, visceral vessel encasement, and two contained rupture PAUs. Suspected etiology included malignancy-related lymphadenitis vs infection. Given suspicion for infected paravisceral saccular aneurysm, high OSR risk, and anatomy requiring urgent visceral branch preservation, the patient underwent successful thoracoabdominal laser FEVAR. This case report was created with the patient's written consent.

## Case report

### Initial presentation

A 68-year-old man with a history of stage 1 bladder cancer undergoing his second year of intravesical Bacillus Calmette-Guérin therapy, hypertension, hyperlipidemia, gout, benign prostatic hyperplasia, and prediabetes presented with a 1-day history of sharp left flank pain. He was tachycardic but hemodynamically stable. Laboratory studies noted leukocytosis of 17.4, hemoglobin of 11.9, creatinine of 0.7, and C-reactive protein of 87. Blood cultures were negative. He denied a prior history of tuberculosis.

**Fig 1:**
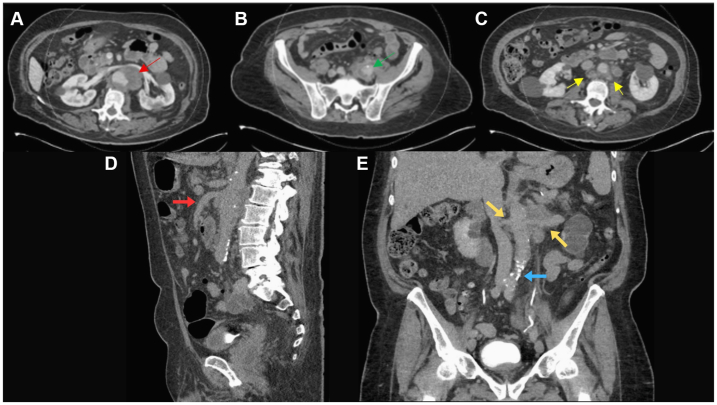
Representative axial images from computed tomography (CT) scans with intravenous (IV) contrast on initial presentation that demonstrated a broad-based outpouching of the suprarenal abdominal aorta measuring 1.3 × 1.4 × 2.1 cm, indicated by the *red arrow***(A)** and a broad-based outpouching from the distal common iliac artery measuring 0.9 × 1.3 × 1.8 cm, indicated by the *green arrow***(B)**. CT imaging also demonstrated significant necrotic lymphadenopathy of the para-aortic lymph nodes **(C)** as well as left iliac chain lymph nodes, indicated by the *yellow arrows*. Coronal images **(D)** demonstrating a broad-based outpouching of the suprarenal abdominal aorta with superior mesenteric artery (SMA) involvement, indicated by the red arrow. CT imaging also demonstrated renal arteries surrounded by dense lymphadenopathy (*yellow arrow*) on a background of enlarged lymph nodes and a narrow bifurcation, indicated by the *blue arrow***(E)**.

A computed tomography (CT) scan demonstrated necrotic para-aortic and iliac lymphadenopathy with encasement and compression of the aorta and left renal vein, and two contained rupture PAUs at the paravisceral aorta and left common iliac artery (Fig 1, *A*-*E*). Lymphadenopathy was suspected to be infectious or malignant, although blood cultures, including acid-fast Bacillus stain, remained negative. Given severe retroperitoneal inflammation, para-aortic necrotic lymphadenopathy, and high concern for rupture, OSR was deemed unsafe. The patient consented to urgent endovascular repair.

### Operative technique

**Fig 2:**
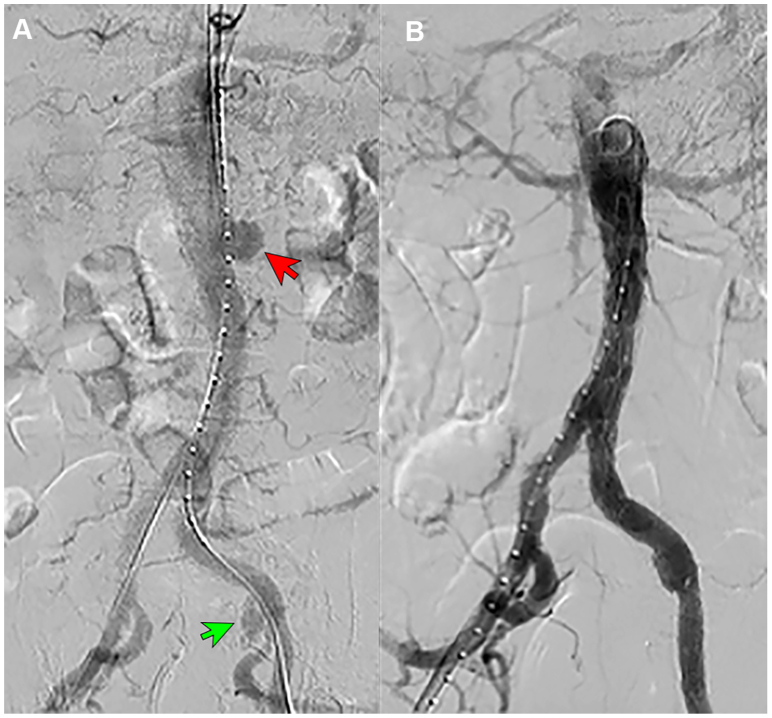
Initial angiogram **(A)** demonstrated the paravisceral penetrating aortic ulcer (PAU) with contained rupture (*red arrow*) and left common iliac artery ulcer with contained rupture (*green arrow*). Completion angiogram **(B)** demonstrated exclusion of both ulcers and ruptures, successful target vessel stenting, and no endoleaks.

Bilateral femoral artery access was upsized to 10F sheaths. After systemic heparinization, a pigtail catheter was advanced to perform an aortogram, confirming contained rupture PAUs adjacent to the left renal artery and superior mesenteric artery (SMA) and at the left common iliac artery near the left hypogastric origin (Fig 2, *A*).

Using a 7F Aptus sheath and 0.035-Glidewire, the right renal artery was cannulated and stented flush at the origin with a 6 × 17 mm VISI-Pro stent (Medtronic). Subsequently, the left renal artery was stented with a 6 × 27 mm VISI-Pro stent, the SMA with an 8 × 27 mm VISI-Pro stent, and the celiac artery with a 9 × 17 mm VISI-Pro stent. Barrel views were saved for each stent to later facilitate laser fenestration. The left internal iliac artery was embolized with an Amplatzer Vascular Plug (St Jude Medical), followed by deployment of an iliac limb to exclude the iliac PAU. A rifampin-soaked EVAR device (AFX2 25-70 mm main body, 16-30 mm bilateral iliac limbs; Endologix) was deployed in the infrarenal aorta with left limb extension given the narrow aortic bifurcation (Fig 1, *E*).

A rifampin-soaked Valiant thoracic stent graft 30 × 26 mm taper by 150 mm device (Medtronic) was deployed across the paravisceral aorta. Using a 2.3-mm laser via an 8.5F Aptus sheath (Medtronic), sequential in vivo fenestrations were created and dilated with a 4-mm Sterling balloon to cannulate and stent the right renal artery (7 × 29 mm VBX), SMA (10 × 29 mm VBX), and left renal artery (7 × 29 mm VBX) with balloon-expandible bridging stents, respectively (W. L. Gore & Associates) and flared at the origin with a plain balloon. Predetermined C-arm angulations were used for barrel views of each target vessel stent to facilitate laser fenestration. Ischemia time for the right renal artery was 5 minutes; for SMA, 15 minutes; and for the left renal artery, 25 minutes. A celiac fenestration was attempted but abandoned due to robust retrograde perfusion through the SMA-gastroduodenal artery arcade. Completion angiography showed exclusion of both PAUs without endoleak (Fig 2, *B*). The activated coagulation time was maintained at 250 seconds. Estimated blood loss was 500 mL. Pedal pulses were present bilaterally upon completion of the case.

### Postoperative course

There was no renal dysfunction after FEVAR. However, on postoperative day 7, the patient developed worsening abdominal pain and a CT scan revealed free air. He underwent exploratory laparotomy, which identified a pinpoint sigmoid perforation with dense inflammatory adhesions, likely secondary to perforated diverticulosis, requiring partial colectomy with 20 cm of sigmoid colon resection, temporary discontinuity, and reanastomosis. There was no ischemia at the perforation site and minimal spillage. Given concern for graft contamination, he completed a 12-week course of oral amoxicillin/clavulanate potassium (Augmentin).

Three months later, he re-presented with malaise, night sweats, and relapsing chest tightness. A CT angiogram demonstrated stable postoperative anatomy without endoleak. Given his history, a plasma microbial cell-free DNA test conducted was positive for *Mycobacterium tuberculosis* complex. He was treated with a 9-month course of rifabutin, isoniazid, pyridoxine, and ethambutol.

At the 9-month follow-up, he presented with a left psoas abscess and left external iliac artery pseudoaneurysm, which were treated with drainage and endovascular stent placement. He recovered without further infectious complications.

### Follow-up

**Fig 3:**
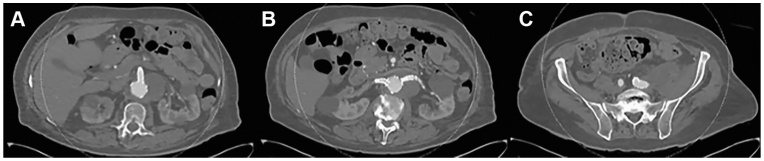
Representative axial images from 6-month follow-up computed tomography (CT) angiogram that demonstrates appropriate exclusion of the paravisceral penetrating aortic ulcer (PAU) and associated contained rupture **(A)** with patent superior mesenteric artery (SMA) **(A)** and bilateral renal arteries **(B)**, as well as exclusion of the left common iliac ulcer and associated contained rupture **(C)**.

One-year surveillance CT angiogram showed durable exclusion of both PAUs, patent visceral stents without evidence of endoleak or graft infection, including no fat stranding or fluid collection, and improved lymphadenopathy (Fig 3).

## Discussion

In this patient, OSR posed unacceptable morbidity due to retroperitoneal inflammation and suspected infection. The literature supports the preferential use of EVAR for mycotic aneurysms, demonstrating improved early survival and fewer perioperative complications when compared with OSR.^4^^,^^10^^,^^11^ EVAR avoids extensive dissection in contaminated or inflamed tissue planes, reducing surgical trauma and physiological stress.^5^

Managing complex aneurysms urgently requires balancing bench-top modification time and intraoperative ischemia time. Although physician-modified endografts maintain perfusion during deployment, they are limited by manual bench-top modification time and potential misalignment in angulated anatomy.^12^ Conversely, laser FEVAR enables rapid repair but necessitates intraoperative ischemia during fenestration. Although SMA ischemia can be minimized to 7 minutes, renal ischemia often extends to 45 to 50 minutes, increasing the risk of renal dysfunction.^12^^,^^13^ Our case demonstrates that minimizing warm ischemia time can decrease this risk.

Laser FEVAR enables emergent visceral revascularization without custom-manufactured devices, allowing rapid repair in urgent settings, with recent studies illustrating technical success and promising patency rates.^12^^,^^13^ Despite the higher risk of infection with EVARs,^4^ this infection risk can be mitigated with prolonged antibiotic courses.^14^

Tuberculosis aortitis is rare, with few published cases. Although historical management favored OSR,^7^^,^^8^ one case suggests endovascular treatment is effective when combined with prolonged antitubercular therapy.^9^ This patient's pathology—necrotizing lymphadenitis with secondary aortic involvement—mirrors known mechanisms of tuberculous aortitis. Rifampin-soaked endografts, although off-label, follow recommendations for infected aortic repairs and may decrease bacterial adherence and suppress early graft colonization.^11^^,^^15^, ^16^, ^17^ Furthermore, a Swedish nationwide study illustrated that shifting from OSR to EVAR for the treatment of mycotic abdominal aortic aneurysms improved early survival without increasing infection-related complications and reoperation rates.^18^ Long-term suppressive therapy likely contributed to the absence of graft infection throughout the follow-up.^10^^,^^11^^,^^14^

Despite colonic perforation, psoas abscess formation, and tuberculous aortitis, the FEVAR remained intact at 6 months and 1 year. This outcome highlights the resilience of FEVAR despite being deployed in infected fields when combined with antibiotic therapy and careful surveillance.

## Conclusions

This case demonstrates that laser FEVAR can provide an effective treatment in high-risk patients with complex aortic pathologies such as tuberculosis aortitis. Combined with rifampin-soaked endografts and prolonged antibiotic therapy, this approach represents a feasible strategy that can achieve meaningful long-term survival in an otherwise life-threatening condition.

## Funding

None.

## Disclosures

None.
